# Vascular endothelium function among male carriers of BRCA 1&2 germline mutation

**DOI:** 10.18632/oncotarget.27118

**Published:** 2019-08-20

**Authors:** Guy Witberg, Eli Lev, Yaara Ber, Tzlil Tabachnik, Sivan Sela, Ira Belo, Dorit Leshem-Lev, David Margel

**Affiliations:** ^1^ Department of Cardiology, Rabin Medical Center, Petach Tikva, Israel; ^2^ The Sackler Faculty of Medicine, Tel Aviv University, Tel Aviv, Israel; ^3^ Department of Urology, Rabin Medical Center, Petach Tikva, Israel; ^4^ Department of Cardiology, Assuta Ashdod University Hospital, Ashdod, Israel; ^5^ Faculty of Medicine, Ben Gurion University, Be'er Sheva, Israel

**Keywords:** BRCA, DNA repair, endothelial progenitor cells, vascular endothelium damage, cardiovascular system

## Abstract

**Background:** Breast cancer susceptibility genes 1&2 (BRCA1&2) mutations hinder DNA-repair. Germline mutations in these genes are known to cause cancer; however, they may have other consequences. In this study we evaluated for the first time, the effect of the BRCA mutations on the vascular endothelium of young healthy males.

**Results:** The study included 82 participants (53 BRCA mutation positive-carriers and 29 negative-carriers). Subjects mean age was 40. There were no significant differences in the baseline characteristics of the two groups. BRCA-carriers had significantly higher levels of EPCs (fraction of CD34+/VEGF or CD133+/VEGF positive-cells) compared to non-carriers of the mutation (median 6.78[1.96,14.48]% vs. 1.46[0.65,6.18]%, *p* < 0.001, and median 7.17[1.70,16.69]% vs. 1.54[0.85,5.10]%, *p* < 0.001, respectively). This difference remained consistent after multivariate adjustment. We did not identify differences in endothelial function, endothelial damage markers and EPCs activity between the two groups.

**Methods:** This was a prospective cohort study to test the association between BRCA status and possible endothelial alterations. The Study population included males, 18-50 years, with no cardiovascular morbidity, who were referred for BRCA screening. We tested the endothelial system by: Endothelial progenitor cells (EPC) production, endothelial function (EndoPAT2000), endothelial damage and related hormonal levels. We stratified the cohort by germline BRCA status and compared measurements between BRCA mutation positive- and negative-carriers.

**Conclusions:** Male BRCA1&2 mutation positive-carriers had increased level of EPCs which may reflect a subclinical accumulative endothelial damage. These novel findings suggest that the effect of mutations in BRCA is not limited to increased cancer risk, but may affect the cardiovascular system.

## INTRODUCTION

Breast Cancer susceptibility genes 1&2 (BRCA1&2) are tumor suppressor genes, involved in DNA-repair and cell cycling [1]. BRCA1&2 mutations are known to increase the risk for breast and ovarian cancer among females. There is also evidence of increased risk for prostate cancer and others among males [2–4].

Limited data suggest that BRCA-mutations are also associated with increased risk for non-cancer related mortality. Mai *et al.* [5]. used an Ashkenazi Jewish cohort from Washington DC to estimate the effect of BRCA1&2 mutations on mortality. They have demonstrated that life expectancy was shorter among BRCA-mutation carriers (5.7 years lower for women, 3.7 for men) compared to age-matched general population.

Physiologically, several common pathways are emerging that link DNA-instability with both cancer and cardiovascular disease. Shukla *et al*. [6] demonstrated a novel role for BRCA1 as a gatekeeper of cardiac-function and survival after ischemia. They showed that in mice, response to stress was deficient in cardiomyocytes lacking BRCA1. These mice had impaired DNA repair mechanisms resulting in activated apoptotic p53-mediated signaling. These pathways are implicated in many cancers as well [7–9]. Similar findings were reported for BRCA2-mutations [10]. In patients with Ataxia-Telangiectasia, a genetic disease with a similar DNA-repair defect, there is evidence of increased risk for premature atherosclerosis [11–15].

One of the earliest manifestations of cardiovascular impairment is “endothelial-dysfunction” [16–19]. Cardiovascular risk factors activate pro-oxidative genes in the endothelium. Thus, generating reactive oxygen species (ROS) leading to release of transcriptional and growth factors, pro-inflammatory cytokines, chemo-attractant substances, and adhesion molecules [15–18, 20].

The primary objective of this study was to compare the endothelial function of positive and negative BRCA-mutation carriers. Furthermore we aimed to explorer possible pathophysiologic pathways that may mediate BRCA’s effect on the cardiovascular system and the vascular endothelium.

## RESULTS

Between 08/07/2015 and 24/07/2017 82 participants (53 BRCA positive and 29 BRCA negative mutation-carriers) were enrolled in the study and underwent genetic testing and endothelial assessment. Baseline characteristics of the participants according to BRCA-mutation status are shown in Table 1. Both groups were similar in terms of age, cardiovascular risk factors, exercise habits, lipid profile and pituitary hormone levels. The mutation positive participants were less likely to be active smokers and more likely to be former smokers then the mutation negative participants. However, there was no difference in pack years between the two groups.

**Table 1:** Baseline characteristics according to BRCA mutation status

	BRCA negative (*n* = 29)	BRCA positive (*n* = 53)	*p*-val
Age, years (avg ± SD)	40.71 ± 6.43	39.09 ± 5.41	0.301
BMI (avg ± SD)	26.23 ± 4.55	26.48 ± 4.05	0.832
Smoking			0.047
Never smoked, *N* (%)	13 (72.2%)	40 (75.5%)	
Former smoker, *N* (%)	2 (11.1%)	12 (22.65)	
Active smoker, *N* (%)	3 (16.7%)	1 (1.9%)	
PY (median, IQR)	0 [0,0.38]	0 [0,0]	0.351
Regular exercise, *N* (%)	12 (66.7%)	32 (60.45)	0.674
Exercise days/week, (median, IQR)	2.5 [0,3.63]	2 [0,3]	0.157
DM, *N* (%)	0 (0%)	1 (1.9%)	1.000
HTN, *N* (%)	3 (16.7%)	2 (3.8%)	0.099
TSH, mlU/L (avg ± SD)	1.89 ± 1.60	1.74 ± 0.72	0.582
FSH, mlU/ml (avg ± SD)	4.17 ± 2.46	4.32 ± 3.12	0.860
LH, mlU/ml (avg ± SD)	4.08 ± 2.27	3.45 ± 1.94	0.260
Prolactin, mcg/l (avg ± SD)	6.58 ± 2.45	5.31 ± 1.94	0.130
Free androgen index (avg ± SD)	45.86 ± 11.84	43.53 ± 14.82	0.549
Testosterone- total, nmol/L (avg ± SD)	18.67 ± 7.17	16.12 ± 5.91	0.142
SHBG, nmol/L (avg ± SD)	42.85 ± 20.97	39.46 ± 15.91	0.477
PSA, ng/ml (avg ± SD)	0.69 ± 0.28	0.6612 ± 0.28	0.675
Cholesterol, mg/dl (avg ± SD)	197.94 ± 38.87	189.63 ± 40.56	0.452
Triglycerides, mg/dl (avg ± SD)	116.78 ± 46.38	146.39 ± 89.24	0.184
HDL, mg/dl (avg ± SD)	46.39 ± 8.41	48.18 ± 14.28	0.619
LDL, mg/dl (avg ± SD)	128.11 ± 35.22	112.98 ± 38.10	0.147

Abbreviations: BMI, body mass index; DM, diabetes mellitus; FSH, follicle stimulating hormone; HDL, high density lipoprotein; HTN, hypertension; LDL, low density lipoprotein; LH, luteinizing hormone; PY, pack years; PSA, prostate specific antigen; SHBG, sex hormone binding globulin; TSH, thyroid stimulating hormone.

### Endothelial function assessment

There was no difference in endothelial function, as assessed by EndoPAT™. The mean LnRHI was 0.717 ± 0.28 in mutation-negative vs. 0.719 ± 0.27 in mutation-positive participants (*p*-value 0.976).

### EPCs assessments

#### Quantitative assessment (Figure 1)

**Figure 1:**
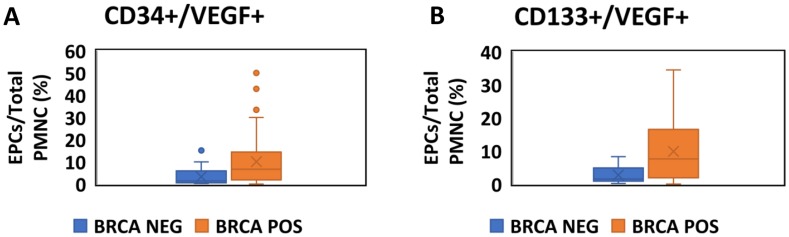
Quantitative assessment of EPCs in BRCA positive carriers and negative carriers. Boxplots comparing the fraction of EPCs as a proportion of total PMNC between BRCA positive (Orange) and Negative (Blue) participants. (**A**) EPCs are identified as CD34+/VEGF+ cells by flow cytometry. (**B**) EPCs are identified as CD133+/VEGF+ cells by flow cytometry.

#### Unadjusted analysis

The fraction of EPC (out of total PMNC) was higher in mutation positive participants either when assessed by CD34+/VEGF+ (median 6.78[1.96,14.48]% vs. 1.46[0.65,6.18]%, *p* < 0.001, Figure 1A) or CD 133+/VEGF+ (median 7.17[1.70,16.69]% vs. 1.54[0.85,5.10]%, *p* < 0.001, Figure 1B), representative FACS data for control and mutation positive carrier patients are shown in Supplementary Figure 1.

### Multivariate adjusted analysis

Using a linear regression model that included age, BMI, smoking status, regular exercise, LDL, HDL, TG, total cholesterol and BRCA mutation status as covariates, BRCA positive-mutation status was associated with an increase of 6.02(SE 2.90)% in CD133+/VEGF+ cells (*P* = 0.024) and of 4.72 (SE 2.49)% in CD34+/VEGF+ cells (*p* = 0.058).

### Qualitative assessment

**Figure 2:**
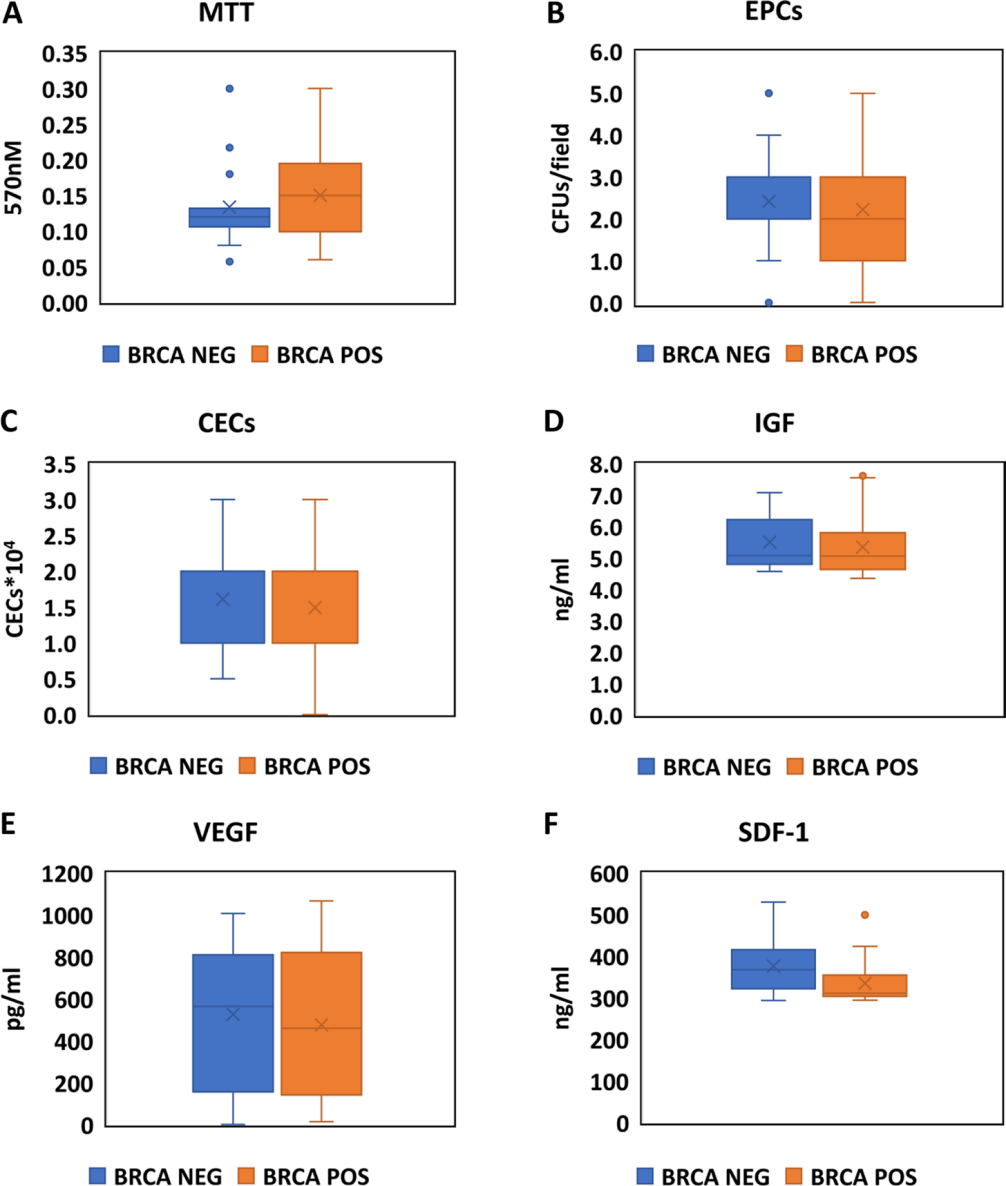
Assessment of EPCs activity, markers of endothelial damage and stimulation for EPCs production in BRCA positive carriers and negative carriers. Boxplots comparing the values of the MTT essay (**A**), CFU/Field (**B**), CECS X 10^4^ (**C**), IGF (**D**), VEGF (**E**) and SDF-1 (**F**) between BRCA positive (Orange) and Negative (Blue) participants.

There was no difference in levels of MTT and CFU between BRCA positive and negative-mutation participants, with either unadjusted analysis or multivariate adjusted linear regression (Figure 2A, 2B and Table 2).

**Table 2:** Association of BRCA mutation status and additional EPC markers

	Unadjusted analysis			Adjusted analysis^*^		
	BRCA+	BRCA−	*p*-val	Beta	SE	*p*-val
CECS X 10^4^	1.25 (1,2)	2 (1,2.25)	0.460	−0.752	0.410	0.188
MTT	0.15 (0.99,0.20)	0.12 (0.10,0.14)	0.239	0.008	0.25	0.753
VEGF	461 (140,821)	565 (226,816)	0.539	−109.9	133.70	0.439
IGF	5.06 (4.63,5.80)	5.08 (4.80,6.22)	0.491	0.10	0.333	0.977
CFU	2 (1,3)	3 (2,3)	0.463	−0.336	0.589	0.574

Values are presented as median (IQR).

Abbreviations: BRCA, breast cancer susceptibility genes; CECS, circulating endothelial cells; VEGF, vascular endothelial growth factor; IGF, insulin like growth factor; CFU, colony forming unit; MTT, 3-(4,5-Dimethylthiazol-2-Yl)-2,5-Diphenyltetrazolium Bromide.

### Endothelial damage assessment

There was no difference in levels of CECX10^4^ between BRCA positive and negative-mutation participants, with either unadjusted analysis or multivariate adjusted linear regression (Figure 2C and Table 2).

### Stimulation for EPCs production

There was no difference in IGF, VEGF or SDF-1 alpha between BRCA positive and negative-mutation participants, with either unadjusted analysis or multivariate adjusted linear regression (Figure 2D–2F and Table 2).

### Other biomarkers

All participants had hsTnT levels that were below 13ng/ml so no comparison between mutation positive and negative participants was performed.

**Figure 3:**
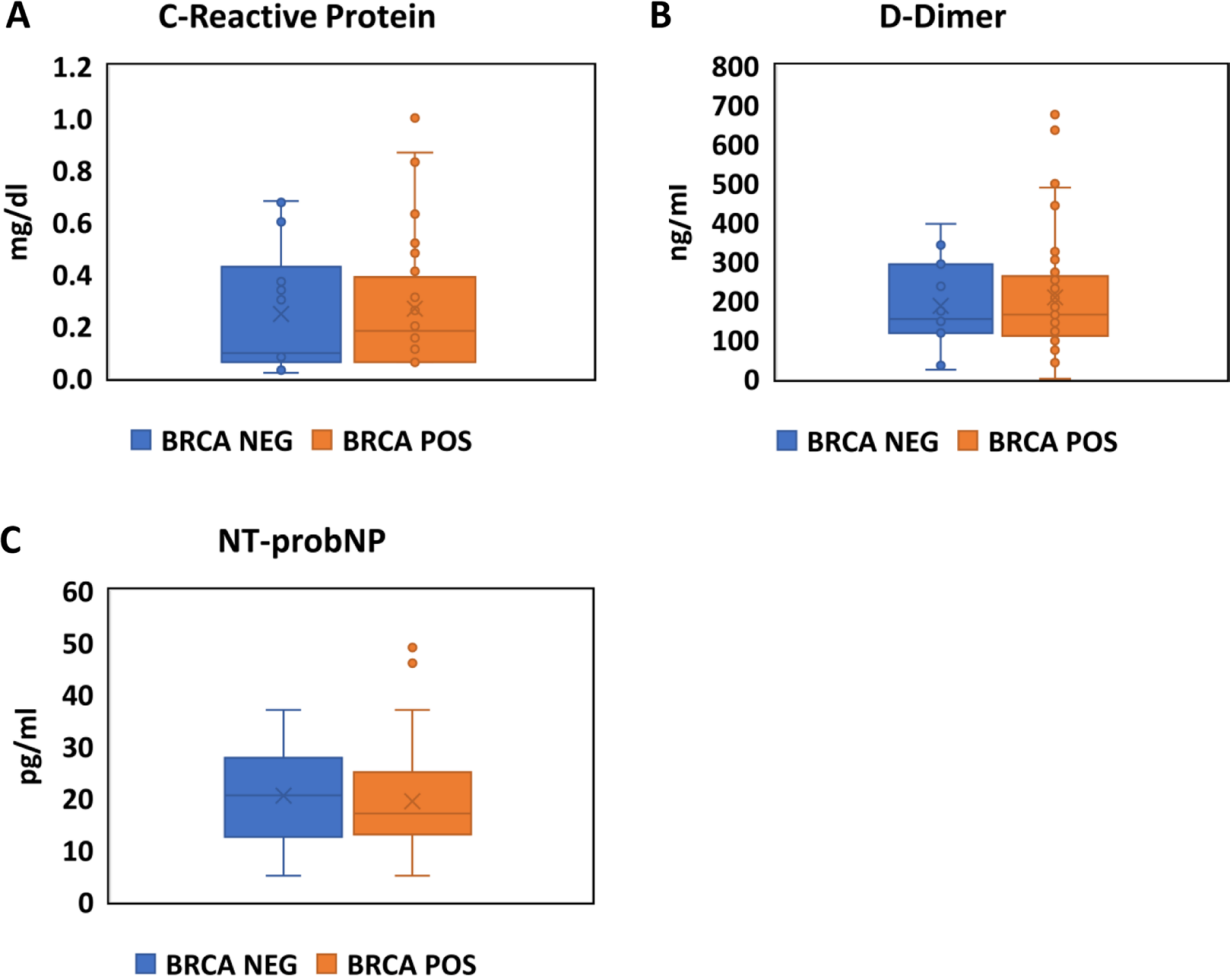
Assessment of inflammation, coagulation and natriuretic peptides in BRCA positive carriers and negative carriers. Boxplots comparing the values CRP (**A**), D-Dimer (**B**) and NTproBNP (**C**) between BRCA positive (Orange) and Negative (Blue) participants.

The results of CRP, D-dimer and NTproBNP levels in BRCA-mutation positive vs. negative participants are shown in Figure 3A–3C and Table 3.

**Table 3:** Association of BRCA mutation status and cardiac biomarkers

	Unadjusted analysis			Adjusted analysis*		
	BRCA+	BRCA−	*p*-val	Beta	SE	*p*-val
CRP, mg/l (median, IQR)	0.21 (0.06,0.47)	0.24 (0.06,42)	252	0.552	0.535	0.308
D-dimer, ng/ml (median, IQR)	164 (110,262)	173 (117,342)	0.628	−54.33	65.02	0.404
NTpro-BNP, pg/ml (median, IQR)	17 (13,25)	23 (14,32)	0.153	−17.65	9.14	0.189

^*^Adjusted for age, BMI, smoking status, regular exercise, LDL, HDL, TG, total cholesterol.

Abbreviations: BRCA, Breast Cancer susceptibility genes; CRP, C-reactive protein; NTproBNP, N-terminal pro b-type Natriuretic Peptide.

There was no significant difference in the levels of CRP, D-dimer or NT-proBNP with either unadjusted (*p* = 0.252, *p* = 0.628 and *p* = 0.153, respectively) or multivariate adjusted analysis using a linear regression model (*p* = 0.308, *p* = 0.404 and *p* = 0.189, respectively) between mutation-positive and negative participants.

## DISCUSSION

The major finding of our study is that male BRCA1&2 positive mutation-carriers show a significantly increased fraction of EPCs, compared to age matched non-carriers. BRCA1&2 mutation status was not associated with a significant differences in endothelial function, overall quantity or viability of endothelial cells or levels of cardiovascular biomarkers (VEGF, hsTnt, NTproBNP D-dimer) and inflammation biomarker (CRP).

Recently, it was shown that DNA-damage and premature vascular aging have a significant role in endothelial-dysfunction [21–23]. Risk factors, such as smoking and diabetes leads to increase in ROS molecules within the atherosclerotic plaque, and induce DNA damage. Genomic instability, such as occurs in BRCA-mutations, can promote endothelial dysfunction and/or cause cell cycle arrest, apoptosis and senescence [24–26].

The hallmarks of BRCA-mutations are genetic instability and DNA-fragmentation thus we hypothesize a link between the two. In women, due to the combination of a high prevalence of cancers at a young age, and cardiovascular diseases at an older age, the effect of BRCA1&2-mutations on cardiovascular morbidity and mortality is difficult to evaluate. However, they might be exposed to increased cardiovascular risk due to exposure to cardiotoxic treatments such as chemotherapy, chest radiotherapy, and/or HER2 directed therapies [27].

In men however, BRCA1&2 related cancers are less prevalent and have better prognosis. Usually, those cancers manifest at older ages than the initial appearances of cardiovascular diseases. Hence, BRCA1&2 related cardiovascular morbidity is important factor for prognosis.

As far as we know, this study is the first to examine possible mechanisms for BRCA1&2’s effect on the endothelial system.

Endothelial progenitor cells (EPCs) regulate vascular integrity by promoting renewal of endothelial cells following injury [24–26]. Specifically, levels of circulating EPCs are lower among patients with chronic stable coronary artery disease (CAD) or with cardiovascular risk factors compared to controls [18]. Moreover, low EPC levels may predict cardiovascular-related events and death from cardiovascular causes [24].

EPCs express cell-surface antigens including CD133, CD34 and vascular endothelial growth factor receptor-2 (VEGFR-2) [28]. EPCs levels, contrary to measuring single serum marker may better estimate the overall function or dysfunction of the endothelial [29, 30]. Our data demonstrates that BRCA1&2-mutations are associated with higher EPC count. Thus, suggesting a pathophysiological link between DNA-repair damage and the endothelial system.

The gold standard to quantify endothelial function is to measure coronary epicardial vaso-reactivity during coronary angiography. It is an invasive procedure that requires specialized equipment and personnel and makes it therefore less suitable in a research setting. The EndoPAT2000 is a non-invasive device that is used to assess endothelial function [31]. We could not find a difference in endothelial function (assessed by EndoPAT), or evidence for endothelial damage (assessed by CECs) according to BRCA1&2-mutations status. We only included subjects younger than 50 years-old. It is possible that at such a young age the BRCA affect is subclinical and may only be realized at an older age.

Although BRCA1&2-mutation carriers had a higher quantity of EPCs then non-carries, there was no difference in the functionality of those cells (assessed by CFU and MTT essay). Similarly, no significant difference was found in factors that stimulate EPCs production (IGF, VEGF) or EPC incorporation into endothelial damage sights (SDF-1 alpha). These results suggest that the difference in EPCs quantity occur due to higher proliferation rates in mutation carriers then in the non-carriers.

Considering all these findings, our proposed hypothesis is that BRCA germline mutations cause an increased damage to endothelial tissue. To compensate there is an increase production of EPCs. This is supported by other reports demonstrating that BRCA is associated with premature ovarian failure and accelerated aging [32–34].

Our study has several limitations. First, our sample size was limited since we used a population that was referred for genetic screening. Second, to avoid bias we only included healthy young males. Therefore functional, clinical differences were not demonstrated. One of the strengths’ of our study was the comprehensive testing of many different aspects of endothelial function. We have found that even in this young cohort without comorbidities the endothelial system is compromised. Moreover, our EPC results were consistent and robust even after multivariate adjustment.

This is one of the first studies to demonstrate a meaningful link between cancer-genes and endothelial-function. Personalized early primary prevention and screening in BRCA1&2-carriers, may reduce the morbidity and mortality associated with cardiovascular disease [35, 36]. Unveiling the physiological mechanism leading to the association between endothelial function and BRCA may in the future lead to novel discoveries of prevention and treatment of both cancer and cardiovascular disease. It may also strengthen the evidence to suggest that cancer is part of a larger metabolic-disease.

## MATERIALS AND METHODS

### Study design

This was a prospective cohort study designed to examine the endothelial-function of young healthy male cohort of BRCA1&2 germline-mutation carriers. Study included male participants that were referred for BRCA-mutation screening due to their family history cancers and BRCA-mutations. Inclusion criteria were: males, age 18-50 years old, definitive results of genetic testing for BRCA-mutation, and willing to sign an informed consent. Exclusion criteria were: history of myocardial infarction, ischemic or hemorrhagic cerebrovascular conditions, arterial embolic or thrombotic events, ischemic heart disease, prior coronary artery revascularization (percutaneous or surgical procedures), or peripheral vascular disease (e. g. claudication, prior vascular surgery/intervention).

### Outcomes

#### Primary outcomes

- Comparison of ∆RHI between BRCA1&2 positive and negative mutation-carriers.
- Difference in fraction of CD34+VEGF+/CD133+VEGF+ cells between BRCA1&2 positive and negative mutation-carriers.

#### Secondary outcomes

- Differences in cardiovascular biomarkers between BRCA positive and negative mutation-carriers.

#### BRCA status assessment

Genetic testing was done for all known 15 founder mutations in Jews, i. e., "The Jewish panel" [37–40] in BRCA1 (ONIM: 113705) and BRCA2 (ONIM: 600185) genes (Supplementary Table 1). Complete DNA-sequencing for all BRCA-mutations was not done due to the fact that these founder mutations constitute 95% of all mutations in Jews [37–40]. The search for mutations was done by Nano-Fluidic Chip 96.96CS (Dynamic Array™ IFC for Genotyping) using the Biomark HD System™ by Fluidigm.

#### Assessment of endothelial function

We used a comprehensive approach to study the endothelial-function with physiological and laboratory assessments. All tests were performed blinded to mutation status:

1. *The Endo-PAT2000 (Itamar Medical Ltd, Keisariya, Israel) -* Endo-PAT2000 is a non-invasive device that measures changes in PAT (Peripheral Arterial Tone) before and after hyperemic response (occluding blood flow through the brachial artery for 5 minutes using an inflatable cuff) [41]. PAT change ratio is normalized to measurements from the contra-lateral arm, which serves as control for non-endothelial dependent systemic effects. The normalized value is termed reactive hyperemia index (RHI) [42]. RHI measure was shown to reflect NO-bioavailability and to correlate with endothelial vasodilator function in coronary artery [43, 44]. RHI value is reduced in patients with established cardiovascular disease and considered to be a predictor for cardiovascular clinical outcomes [45, 46].

2. *Endothelial Progenitor Cells (EPCs) -* are early stem cells that play an important role in the regeneration of the endothelial lining of blood vessels.

### a. Quantitative assessment of EPCs

#### Isolation of mononuclear cells

Samples were centrifuged using Ficoll density gradient centrifugation. Peripheral mononuclear cells (PMNCs) were isolated and washed with phosphate-buffered saline after red cell lysis.

#### Flow cytometry

We incubated PMNCs with monoclonal antibodies for FITC labelled VEGFR-2 (R&D, Minneapolis, USA), CD45-CYT5.5 (Dako, Denmark), and either CD133 (PE-labelled), or CD34 (PE-labelled, Miltenyi Biotech, Auburn, CA, USA). Antibodies with identical isotype were used as controls. Following incubation, we washed the cells with phosphate-buffered saline and analyzed by flow cytometer (FACSCalibur, Becton Dickinson) as described before [47]. Each analysis included 100,000 events after selection for CD45-positive cells (including low-intensity CD45+ cells) and exclusion of debris. In the next step, gated CD34 or CD133-positive cells were examined for the expression of VEGFR-2. Results are presented as the percentage of PMNCs co-expressing VEGF+/CD133+, or VEGF+/CD34+. Analyses were performed in duplicates.

### b. Functional assessment of endothelial progenitor cells

#### Colony forming unit (CFU) assay

We re-suspended isolated PMNCs with Medium 199 (Invitrogen, Carlsbad, CA, USA), supplemented with 20% fetal calf serum (Gibco BRL Life Tech, Gaithersburg, MD, USA). Then, cells were plated on six-well plates coated with human fibronectin (4 × 10^6^ cells per well). After 7 days, we counted the EPCs colonies. EPC colony was defined as a cluster of at least 100 flat cells surrounding a cluster of rounded cells [48]. Results are displayed as mean number of CFUs per field.

#### MTT assay

MTT (3-[4,5-dimethylthiazol-2-yl]-2,5-diphenyl tetrazolium bromide) measures mitochondrial activity in living cells and used to evaluate viability of cultured cells. The assay was performed as previously described [47, 49]. After 7 days of culture, We added 1 mg/ml MTT (Sigma, St. Louis, USA) to the EPCs medium, and incubated for additional 3-4 hours. After incubation, the medium was removed and the cells were solubilized in isopropanol. The amount of dye, which was released from the cells and correlated to the number of living cells, was measured with a spectrophotometer at 570nm and subtracted background at 690nm. An increase in the number of viable cells results in an increase in the amount of MTT formed and, therefore, in absorbance. Results were corrected for the number of EPC CFUs in each group.

### Assessment of endothelial damage

#### Evaluation of circulating endothelial cells (CECs)

Detection and quantification of CECs were performed using monodispersed magnetizable particles (Dynabeads CELLection Pan Mouse IgG kit; Invitrogen, Carlsbad, CA, USA) as previously described [50, 51]. Typically, 100 ml of bead suspension was noncovalently coated with 1.5 mg/ml of anti CD 146 (Novus Biologicals, Littleton, CO, USA, http://www.novusbio.com), an endothelial cell-specific monoclonal antibody. Beads and target cells were incubated for 1.5 h at 4° C on a rotator. Separation of beads and rosetted cells from the blood samples required a minimum of a 1min exposure to the magnet. Three washes were performed in this device to completely remove nonrosetted cells. After the third wash, rosetted cells were recovered in a 50-μl solution of acridine orange (a vital fluorescent dye at final concentration of 5 μg/ml in PBS), and observations were made in a hemacytometer under both white and fluorescent blue excitation using fluorescence microscopy.

#### Assessment of chemokine/ growth factors in the plasma

Plasma samples were stored at −70° C and later evaluated for vascular endothelial growth factor (VEGF), Insulin-like Growth Factor (IGF) and Stromal Cell-Derived Factor 1 (SDF-1) alpha, using ELISA assay. Kits were used according to the manufacturer’s instructions (RayBiotech, Inc).

#### Assessment of cardiovascular biomarkers

Four biomarkers associated with different pathophysiologic pathways that affect the cardiovascular system were measured: High sensitive Troponin T (hsTnT) – to assess myocardial injury, C Reactive-Protein (CRP) – to assess inflammation, D-dimer – to assess activation of the coagulation system, N-terminal pro-brain natriuretic peptide (NTproBNP) – to assess myocardial strain. All assays were performed by ELISA, according to manufacturers’ instructions.

### Statistical analysis

Baseline characteristics are reported as numbers and percentages for categorical variables, and means ±SD or as medians (interquartile range) for continuous variables as appropriate. The characteristics were compared between BRCA-carriers and controls using the paired *t*-test or the Mann-Whitney *U*-test for continuous variables as appropriate, and Fisher’s exact test for categorical variables.

Outcome measures are reported as means ± SD or as medians (interquartile range) as appropriate and were compared between BRCA carriers and controls using the paired *t*-test or the Mann-Whitney *U*-test as appropriate.

In addition, we performed an adjusted analysis by fitting a linear regression model for prediction of each outcome measure that included all relevant baseline characteristics as well as BRCA1&2-mutation status.

### Sample size calculation

Our primary outcome was RHI (a continuous variable). Previous studies have shown that endothelial function (assessed by RHI) was normally distributed with a standard deviation of 0.42 and that a clinically meaningful difference in endothelial function is ΔLnRHI of 0.26 [31]. Based on these assumptions, in order to have an 80% power to detect a clinically significant difference in RHI at a confidence level of 95%, a sample size of 82 patients was required. Allowing for 10% attrition of recruited patients, we aimed to reach a sample size of 90 patients. The actual sample size was 82 patients.

### Ethical aspects, consent and confidentiality

The study was approved by the Rabin Medical-Center institutional ethics review board, in accordance to the Declaration of Helsinki. All participants received detailed verbal and printed information about the study, and signed a written informed consent form before enrollment in the study.

## SUPPLEMENTARY MATERIALS



## References

[1] Yoshida K, Miki Y Role of BRCA1 and BRCA2 as regulators of DNA repair, transcription, and cell cycle in response to DNA damage. Cancer Sci. 2004; 95:866–71. 10.1111/j.1349-7006.2004.tb02195.x. 15546503 PMC11159131

[2] King MC, Marks JH, Mandell JB, and New York Breast Cancer Study Group Breast and ovarian cancer risks due to inherited mutations in BRCA1 and BRCA2. Science. 2003; 302:643–46. 10.1126/science.1088759. 14576434

[3] Ford D, Easton DF, Bishop DT, Narod SA, Goldgar DE, and Breast Cancer Linkage Consortium Risks of cancer in BRCA1-mutation carriers. Lancet. 1994; 343:692–95. 10.1016/S0140-6736(94)91578-4. 7907678

[4] Thompson D, Easton D, and Breast Cancer Linkage Consortium Variation in cancer risks, by mutation position, in BRCA2 mutation carriers. Am J Hum Genet. 2001; 68:410–19. 10.1086/318181. 11170890 PMC1235274

[5] Mai PL, Chatterjee N, Hartge P, Tucker M, Brody L, Struewing JP, Wacholder S Potential excess mortality in BRCA1/2 mutation carriers beyond breast, ovarian, prostate, and pancreatic cancers, and melanoma. PLoS One. 2009; 4:e4812. 10.1371/journal.pone.0004812. 19277124 PMC2652075

[6] Shukla PC, Singh KK, Quan A, Al-Omran M, Teoh H, Lovren F, Cao L, Rovira II, Pan Y, Brezden-Masley C, Yanagawa B, Gupta A, Deng CX, et al BRCA1 is an essential regulator of heart function and survival following myocardial infarction. Nat Commun. 2011; 2:593. 10.1038/ncomms1601. 22186889 PMC3247816

[7] Cho Y, Gorina S, Jeffrey PD, Pavletich NP Crystal structure of a p53 tumor suppressor-DNA complex: understanding tumorigenic mutations. Science. 1994; 265:346–55. 10.1126/science.8023157. 8023157

[8] Kern SE, Kinzler KW, Bruskin A, Jarosz D, Friedman P, Prives C, Vogelstein B Identification of p53 as a sequence-specific DNA-binding protein. Science. 1991; 252:1708–11. 10.1126/science.2047879. 2047879

[9] May P, May E Twenty years of p53 research: structural and functional aspects of the p53 protein. Oncogene. 1999; 18:7621–36. 10.1038/sj.onc.1203285. 10618702

[10] Singh KK, Shukla PC, Quan A, Desjardins JF, Lovren F, Pan Y, Garg V, Gosal S, Garg A, Szmitko PE, Schneider MD, Parker TG, Stanford WL, et al BRCA2 protein deficiency exaggerates doxorubicin-induced cardiomyocyte apoptosis and cardiac failure. J Biol Chem. 2012; 287:6604–14. 10.1074/jbc.M111.292664. 22157755 PMC3325595

[11] Uryga A, Gray K, Bennett M DNA Damage and Repair in Vascular Disease. Annu Rev Physiol. 2016; 78:45–66. 10.1146/annurev-physiol-021115-105127. 26442438

[12] Mercer JR, Cheng KK, Figg N, Gorenne I, Mahmoudi M, Griffin J, Vidal-Puig A, Logan A, Murphy MP, Bennett M DNA-damage links mitochondrial dysfunction to atherosclerosis and the metabolic syndrome. Circ Res. 2010; 107:1021–31. 10.1161/CIRCRESAHA.110.218966. 20705925 PMC2982998

[13] Mercer JR, Yu E, Figg N, Cheng KK, Prime TA, Griffin JL, Masoodi M, Vidal-Puig A, Murphy MP, Bennett MR The mitochondria-targeted antioxidant MitoQ decreases features of the metabolic syndrome in ATM+/-/ApoE-/- mice. Free Radic Biol Med. 2012; 52:841–49. 10.1016/j.freeradbiomed.2011.11.026. 22210379

[14] Jia L, Zhang W, Ma Y, Chen B, Liu Y, Piao C, Wang Y, Yang M, Liu T, Zhang J, Li T, Nie S, Du J Haplodeficiency of Ataxia Telangiectasia Mutated Accelerates Heart Failure After Myocardial Infarction. J Am Heart Assoc. 2017; 6:e006349. 10.1161/JAHA.117.006349. 28724653 PMC5586323

[15] Quyyumi AA, Dakak N, Andrews NP, Husain S, Arora S, Gilligan DM, Panza JA, Cannon RO 3rd Nitric oxide activity in the human coronary circulation. Impact of risk factors for coronary atherosclerosis. J Clin Invest. 1995; 95:1747–55. 10.1172/JCI117852. 7706483 PMC295695

[16] Quyyumi AA Endothelial function in health and disease: new insights into the genesis of cardiovascular disease. Am J Med. 1998; 105:32S–39S. 10.1016/S0002-9343(98)00209-5. 9707266

[17] Hill JM, Zalos G, Halcox JP, Schenke WH, Waclawiw MA, Quyyumi AA, Finkel T Circulating endothelial progenitor cells, vascular function, and cardiovascular risk. N Engl J Med. 2003; 348:593–600. 10.1056/NEJMoa022287. 12584367

[18] Anderson TJ, Uehata A, Gerhard MD, Meredith IT, Knab S, Delagrange D, Lieberman EH, Ganz P, Creager MA, Yeung AC, Selwyn AP Close relation of endothelial function in the human coronary and peripheral circulations. J Am Coll Cardiol. 1995; 26:1235–41. 10.1016/0735-1097(95)00327-4. 7594037

[19] Hadi HA, Carr CS, Al Suwaidi J Endothelial dysfunction: cardiovascular risk factors, therapy, and outcome. Vasc Health Risk Manag. 2005; 1:183–98. 17319104 PMC1993955

[20] Cai H, Harrison DG Endothelial dysfunction in cardiovascular diseases: the role of oxidant stress. Circ Res. 2000; 87:840–44. 10.1161/01.RES.87.10.840. 11073878

[21] Werner N, Kosiol S, Schiegl T, Ahlers P, Walenta K, Link A, Böhm M, Nickenig G Circulating endothelial progenitor cells and cardiovascular outcomes. N Engl J Med. 2005; 353:999–1007. 10.1056/NEJMoa043814. 16148285

[22] Urbich C, Dimmeler S Endothelial progenitor cells: characterization and role in vascular biology. Circ Res. 2004; 95:343–53. 10.1161/01.RES.0000137877.89448.78. 15321944

[23] Garg R, Tellez A, Alviar C, Granada J, Kleiman NS, Lev EI The effect of percutaneous coronary intervention on inflammatory response and endothelial progenitor cell recruitment. Catheter Cardiovasc Interv. 2008; 72:205–09. 10.1002/ccd.21611. 18651648

[24] Andreassi MG DNA damage, vascular senescence and atherosclerosis. J Mol Med (Berl). 2008; 86:1033–43. 10.1007/s00109-008-0358-7. 18563380

[25] Wang JC, Bennett M Aging and atherosclerosis: mechanisms, functional consequences, and potential therapeutics for cellular senescence. Circ Res. 2012; 111:245–59. 10.1161/CIRCRESAHA.111.261388. 22773427

[26] Herbert KE, Mistry Y, Hastings R, Poolman T, Niklason L, Williams B Angiotensin II-mediated oxidative DNA damage accelerates cellular senescence in cultured human vascular smooth muscle cells via telomere-dependent and independent pathways. Circ Res. 2008; 102:201–08. 10.1161/CIRCRESAHA.107.158626. 17991883 PMC2861985

[27] Gast KC, Viscuse PV, Nowsheen S, Haddad TC, Mutter RW, Wahner Hendrickson AE, Couch FJ, Ruddy KJ Cardiovascular Concerns in BRCA1 and BRCA2 Mutation Carriers. Curr Treat Options Cardiovasc Med. 2018; 20:18. 10.1007/s11936-018-0609-z. 29497862

[28] Peichev M, Naiyer AJ, Pereira D, Zhu Z, Lane WJ, Williams M, Oz MC, Hicklin DJ, Witte L, Moore MA, Rafii S Expression of VEGFR-2 and AC133 by circulating human CD34(+) cells identifies a population of functional endothelial precursors. Blood. 2000; 95:952–58. 10648408

[29] Schmidt-Lucke C, Rössig L, Fichtlscherer S, Vasa M, Britten M, Kämper U, Dimmeler S, Zeiher AM Reduced number of circulating endothelial progenitor cells predicts future cardiovascular events: proof of concept for the clinical importance of endogenous vascular repair. Circulation. 2005; 111:2981–87. 10.1161/CIRCULATIONAHA.104.504340. 15927972

[30] Dimmeler S, Zeiher AM Vascular repair by circulating endothelial progenitor cells: the missing link in atherosclerosis? J Mol Med (Berl). 2004; 82:671–77. 10.1007/s00109-004-0580-x. 15322703

[31] McCrea CE, Skulas-Ray AC, Chow M, West SG Test-retest reliability of pulse amplitude tonometry measures of vascular endothelial function: implications for clinical trial design. Vasc Med. 2012; 17:29–36. 10.1177/1358863X11433188. 22363016 PMC3513268

[32] Oktay K, Turan V, Titus S, Stobezki R, Liu L BRCA Mutations, DNA Repair Deficiency, and Ovarian Aging. Biol Reprod. 2015; 93:67. 10.1095/biolreprod.115.132290. 26224004 PMC4710189

[33] Uziel O, Yerushalmi R, Zuriano L, Naser S, Beery E, Nordenberg J, Lubin I, Adel Y, Shepshelovich D, Yavin H, Ben Aharon I, Pery S, Rizel S, et al BRCA1/2 mutations perturb telomere biology: characterization of structural and functional abnormalities *in vitro* and *in vivo* . Oncotarget. 2016; 7:2433–54. 10.18632/oncotarget.5693. 26515461 PMC4823046

[34] Cho NW, Lampson MA, Greenberg RA *In vivo* imaging of DNA double-strand break induced telomere mobility during alternative lengthening of telomeres . Methods. 2017; 114:54–59. 10.1016/j.ymeth.2016.07.010. 27491801 PMC5378164

[35] Piepoli MF, Hoes AW, Agewall S, Albus C, Brotons C, Catapano AL, Cooney MT, Corrà U, Cosyns B, Deaton C, Graham I, Hall MS, Hobbs FD, et al, and ESC Scientific Document Group 2016 European Guidelines on cardiovascular disease prevention in clinical practice: The Sixth Joint Task Force of the European Society of Cardiology and Other Societies on Cardiovascular Disease Prevention in Clinical Practice (constituted by representatives of 10 societies and by invited experts)Developed with the special contribution of the European Association for Cardiovascular Prevention & Rehabilitation (EACPR). Eur Heart J. 2016; 37:2315–81. 10.1093/eurheartj/ehw106. 27222591 PMC4986030

[36] Liu K, Daviglus ML, Loria CM, Colangelo LA, Spring B, Moller AC, Lloyd-Jones DM Healthy lifestyle through young adulthood and the presence of low cardiovascular disease risk profile in middle age: the Coronary Artery Risk Development in (Young) Adults (CARDIA) study. Circulation. 2012; 125:996–1004. 10.1161/CIRCULATIONAHA.111.060681. 22291127 PMC3353808

[37] Abeliovich D, Kaduri L, Lerer I, Weinberg N, Amir G, Sagi M, Zlotogora J, Heching N, Peretz T The founder mutations 185delAG and 5382insC in BRCA1 and 6174delT in BRCA2 appear in 60% of ovarian cancer and 30% of early-onset breast cancer patients among Ashkenazi women. Am J Hum Genet. 1997; 60:505–14. 9042909 PMC1712523

[38] Lerer I, Wang T, Peretz T, Sagi M, Kaduri L, Orr-Urtreger A, Stadler J, Gutman H, Abeliovich D The 8765delAG mutation in BRCA2 is common among Jews of Yemenite extraction. Am J Hum Genet. 1998; 63:272–74. 10.1086/301924. 9634522 PMC1377245

[39] Shiri-Sverdlov R, Gershoni-Baruch R, Ichezkel-Hirsch G, Gotlieb WH, Bruchim Bar-Sade R, Chetrit A, Rizel S, Modan B, Friedman E The Tyr978X BRCA1 Mutation in Non-Ashkenazi Jews: Occurrence in High-Risk Families, General Population and Unselected Ovarian Cancer Patients. Community Genet. 2001; 4:50–55. 11493753 10.1159/000051156

[40] Sagi M, Eilat A, Ben Avi L, Goldberg Y, Bercovich D, Hamburger T, Peretz T, Lerer I Two BRCA1/2 founder mutations in Jews of Sephardic origin. Fam Cancer. 2011; 10:59–63. 10.1007/s10689-010-9395-9. 21063910

[41] Endothelial function assessment (Endo PAT) Trachea button - Product information Itamar Medical Ltd, Israel Available at http://www.itamar-medical.com (Accessed 1 Aug 2017).

[42] Flammer AJ, Anderson T, Celermajer DS, Creager MA, Deanfield J, Ganz P, Hamburg NM, Lüscher TF, Shechter M, Taddei S, Vita JA, Lerman A The assessment of endothelial function: from research into clinical practice. Circulation. 2012; 126:753–67. 10.1161/CIRCULATIONAHA.112.093245. 22869857 PMC3427943

[43] Nohria A, Gerhard-Herman M, Creager MA, Hurley S, Mitra D, Ganz P Role of nitric oxide in the regulation of digital pulse volume amplitude in humans. J Appl Physiol (1985). 2006; 101:545–48. 10.1152/japplphysiol.01285.2005. 16614356

[44] Bonetti PO, Pumper GM, Higano ST, Holmes DR Jr, Kuvin JT, Lerman A Noninvasive identification of patients with early coronary atherosclerosis by assessment of digital reactive hyperemia. J Am Coll Cardiol. 2004; 44:2137–41. 10.1016/j.jacc.2004.08.062. 15582310

[45] Bonetti PO, Pfisterer M, Lerman A Attenuation of Digital Reactive Hyperemia in Patients with Early and Advanced Coronary Artery Disease. J Am Coll Cardiol. 2005; 45:407A.

[46] Rubinshtein R, Kuvin JT, Soffler M, Lennon RJ, Lavi S, Nelson RE, Pumper GM, Lerman LO, Lerman A Assessment of endothelial function by non-invasive peripheral arterial tonometry predicts late cardiovascular adverse events. Eur Heart J. 2010; 31:1142–48. 10.1093/eurheartj/ehq010. 20181680

[47] Lev EI, Leshem-Lev D, Mager A, Vaknin-Assa H, Harel N, Zimra Y, Bental T, Greenberg G, Dvir D, Solodky A, Assali A, Battler A, Kornowski R Circulating endothelial progenitor cell levels and function in patients who experienced late coronary stent thrombosis. Eur Heart J. 2010; 31:2625–32. 10.1093/eurheartj/ehq184. 20543191

[48] Fadini GP, Sartore S, Schiavon M, Albiero M, Baesso I, Cabrelle A, Agostini C, Avogaro A Diabetes impairs progenitor cell mobilisation after hindlimb ischaemia-reperfusion injury in rats. Diabetologia. 2006; 49:3075–84. 10.1007/s00125-006-0401-6. 17072586

[49] Chen JZ, Zhu JH, Wang XX, Zhu JH, Xie XD, Sun J, Shang YP, Guo XG, Dai HM, Hu SJ Effects of homocysteine on number and activity of endothelial progenitor cells from peripheral blood. J Mol Cell Cardiol. 2004; 36:233–39. 10.1016/j.yjmcc.2003.10.005. 14871551

[50] Sambuceti G, Morbelli S, Vanella L, Kusmic C, Marini C, Massollo M, Augeri C, Corselli M, Ghersi C, Chiavarina B, Rodella LF, L’Abbate A, Drummond G, et al Diabetes impairs the vascular recruitment of normal stem cells by oxidant damage, reversed by increases in pAMPK, heme oxygenase-1, and adiponectin. Stem Cells. 2009; 27:399–407. 10.1634/stemcells.2008-0800. 19038792 PMC2729677

[51] Abraham NG, Rezzani R, Rodella L, Kruger A, Taller D, Li Volti G, Goodman AI, Kappas A Overexpression of human heme oxygenase-1 attenuates endothelial cell sloughing in experimental diabetes. Am J Physiol Heart Circ Physiol. 2004; 287:H2468–77. 10.1152/ajpheart.01187.2003. 15284058

