# Supplementary material for: Vascular endothelium function among male carriers of BRCA 1&2 germline mutation
Source: Oncotarget. 2019 Aug 20;10(49):5041–51. doi: 10.18632/oncotarget.27118 (PMC6707947; doi:10.18632/oncotarget.27118)
Supplement: Supplementary file 1 [file oncotarget-10-5041-s001.pdf]

Vascular endothelium function among male carriers of BRCA 1&2 germline mutation

SUPPLEMENTARY MATERIALS

Supplementary Table 1: 15 known BRCA 1&2 founder mutations in Jews

BRCA Mutation tested	
**BRCA1: nm_07284.2**	**Known clinical name (according to GenBank U14680.1)**
p. E23VfsX17, c.68_69delAG	185delAG
P. Q1756PfsX74, c.5266dupC	5382insC
p. E1346KfsX20, C.4035delA	4153delA
p. A1708E, c.5123C>A	A1708E
p. Y978×, c.2934T>G	Y978X
p. C61G, c.181T>G	300T>G
p. C328×, c.981delAT	1100delAT
p. E720×, c.2158G>T	E720X
p. W1508×, c.4524>A	W1508X
**BRCA2: nm_000059.3**	**Known clinical name (according to GenBank U43746)**
p. S1982RfsX22, c.5946delT	6174delT
p. E2846GfsX22, c.8537_8538delAG	8765delAG
p. V1283KfsX2, c.3847_3848delGT	4075delGT
c.67+1G>A	IVS2+IG>A
p. R2336P, c.7007G>C	R2336P
p. E1646QfsX23, c.4936_4939delGAAA	5164del4

This table is based on data from the Cancer Information Core Database. http://research.nhgri.nih.gov/projects/bic/.

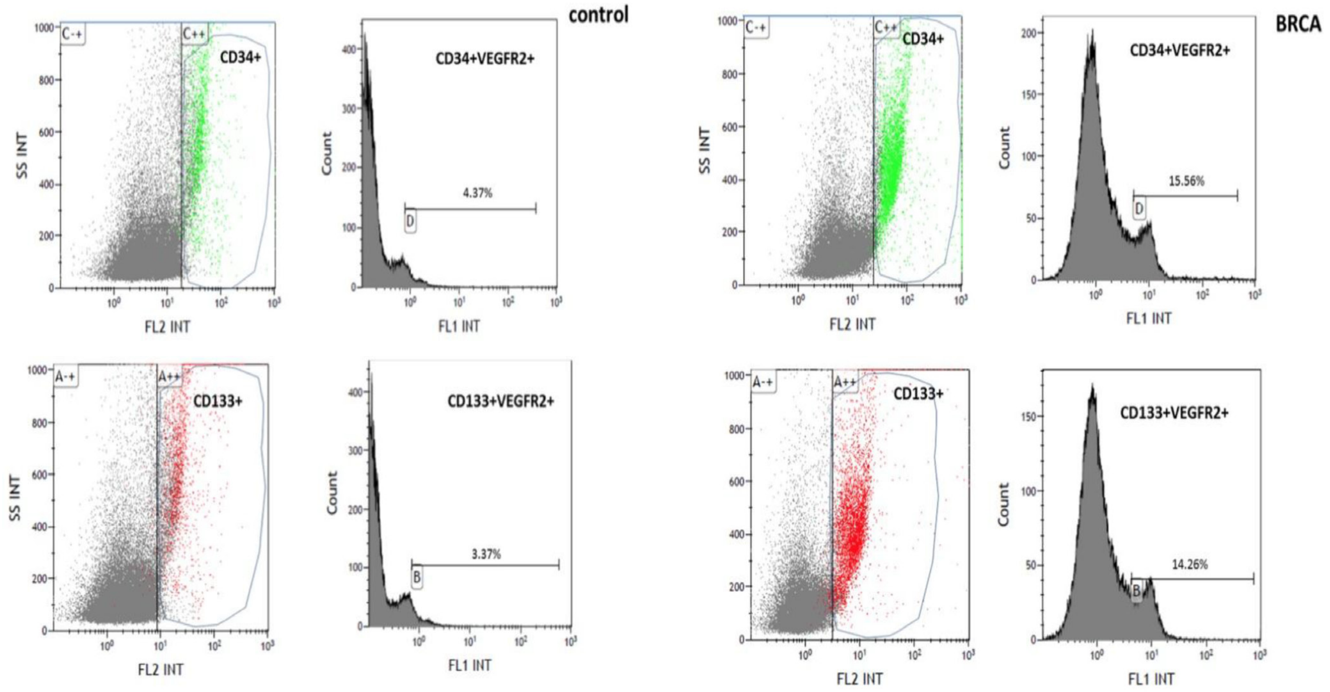

Supplementary Figure 1: Representative FACS analysis for CD34+/VEGF-R2+ and CD133+/VEGF-R2+ in control (left) and BRCA mutation positive patient (right).
